# Supplementary material for: Evidence supported by Mendelian randomization: impact on inflammatory factors in knee osteoarthritis
Source: Front Med (Lausanne). 2024 May 28;11:1382836. doi: 10.3389/fmed.2024.1382836 (PMC11165061; doi:10.3389/fmed.2024.1382836)
Supplement: Supplementary file 1 [file Data_Sheet_1.ZIP › data/GCST90274824_5e6/824.forest.pdf]

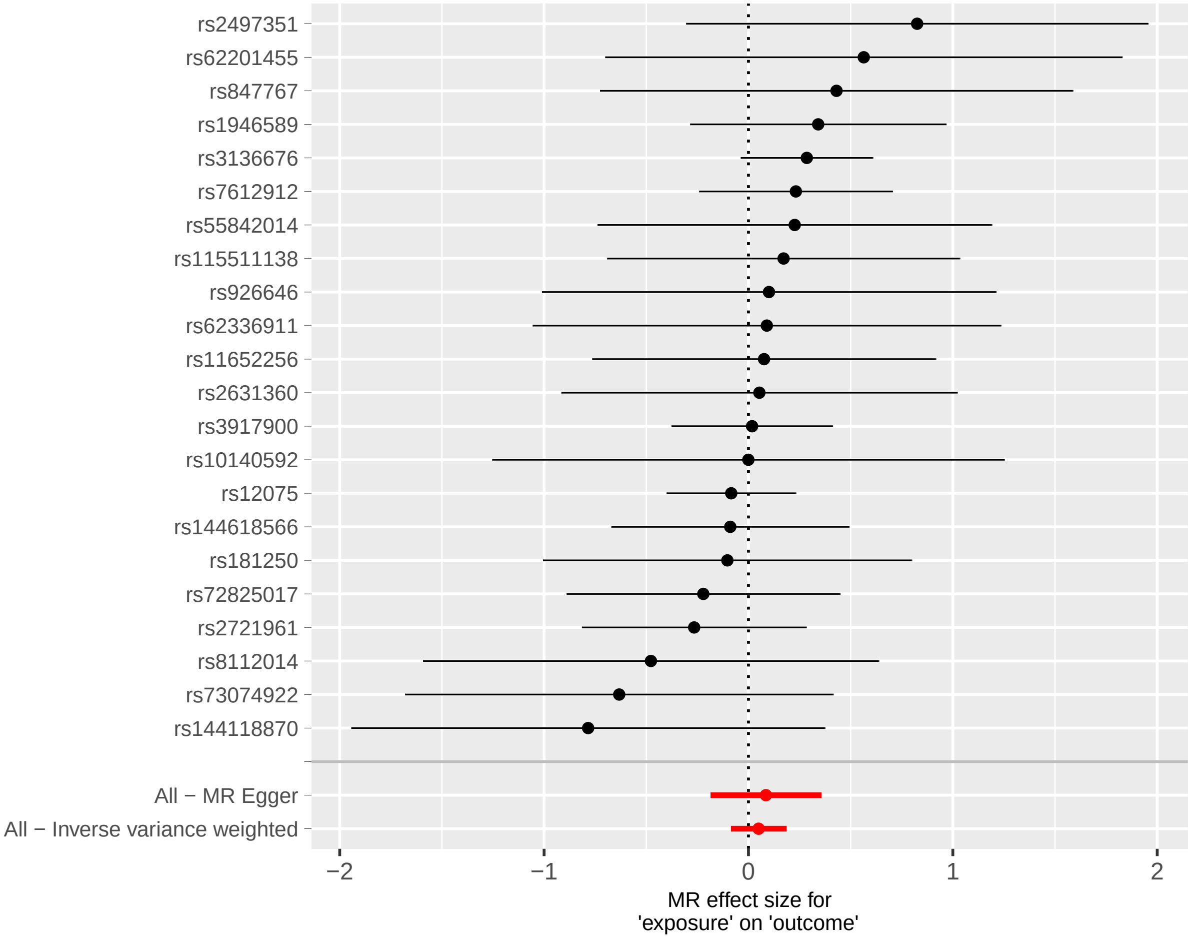
rs2497351
rs62201455
rs847767
rs1946589
rs3136676
rs7612912
rs55842014
rs115511138
rs926646
rs62336911
rs11652256
rs2631360
rs3917900
rs10140592
rs12075
rs144618566
rs181250
rs72825017
rs2721961
rs8112014
rs73074922
rs144118870
All − MR Egger
All − Inverse variance weighted
−2
−1
0
1
2
MR effect size for
'exposure' on 'outcome'
